# Supplementary material for: Multiphysics simulation of magnetoelectric micro core-shells for wireless cellular stimulation therapy via magnetic temporal interference
Source: PLoS One. 2024 Jan 25;19(1):e0297114. doi: 10.1371/journal.pone.0297114 (PMC10834063; doi:10.1371/journal.pone.0297114)
Supplement: Attachment — Submitted filename: Support Letter--PLOS-ONE.docx [file pone.0297114.s001.docx]

Support Letter for Submission titled:

**Multiphysics Simulation of Magnetoelectric Microdevices for Wireless Cellular Stimulation Therapy via Magnetic Temporal Interference**

Dear editors,

We recently submitted our work to PLOS-ONE and have since received the review letter. We sincerely appreciate the valuable feedback provided. The comments were instrumental in helping us identify and address issues within our paper. Our original submission number was PONE-D-23-13385. We have thoroughly revised the manuscript and are now resubmitting it. Below, please find a detailed response to each review comment and the corresponding changes made in the current version.

**Reviewer #1:**

*We would like to express our sincere gratitude for the time and effort you dedicated to reviewing our paper. Your insightful comments have been invaluable. We have made the necessary modifications to the paper and have thoroughly addressed each of your comments. Below, you will find a detailed breakdown of the review comments alongside our responses. We hope that this revised version meets your expectations and further elevates the quality of the research presented.*

**Comment 1**: -It is not clear why there is the need to use the magnetic temporal interference (MTI) signaling. There is no comparison with the case in which it is not used. What is the main advantage of using it? why it is so needed? why the use of two MF sources should be better than just one?

**Reply:** The magnetic temporal interference is necessary to demonstrate the non-linear effect of magnetostrictive materials in signal multiplication and subsequent wave demodulation. Demodulation is key in converting the applied high-frequency field components into low-frequency energy counterparts, facilitating electrical interactions with biological models. Consequently, our system utilizes two frequencies with a slight shift; this frequency shift serves as the system's output for cell stimulation. Additionally, the use of high frequency is necessary to create the multimode resonances with precise hotspots for stimulation control. In Fig 7 and 8, we validate that the demodulation source is the magnetostrictive material and not the electromagnetic interference.

**Comment 2**: -Why did you choose these specific frequencies? What will be the effect of other frequencies?
**Reply:** The magnetostrictive core is notably large. This results in multimodal resonances across various frequencies, pinpointed by the eigenmode solver. Some of these modes resonate with minimal loss, and we anticipate the most significant deformation at these frequencies. This deformation acts as the primary source of depolarization on the piezo layer, which we aim to maximize for effective cell stimulation. Ideally, the two selected frequencies should differ by hundreds of Hz to interact optimally with cell functions. We've opted for a greater frequency difference to simplify the simulation process and enhance memory efficiency.

In section 3.2 (from lines 260 - 265), we have explained the choice of frequency selection. Please note that we have tried simulation with lower frequencies and lesser difference between the selected frequencies. Even with a 2D simulation case and simulating only half of the geometry (since the core shell structure is axis symmetric), the simulation near to 100 hours to run, resulting in a run-out of memory in the system. For example, if we select two frequencies so that the difference between them is in 100s of Hz, we need to stretch one cycle of simulation time of ~10 -- 100s of ms. To run the same and see the input and the difference frequencies, the step size must also be very less (in the order of ~100s of micro-seconds). To reduce these complexities, and to show the working of the hypothesis (non-linear frequency demodulation), we selected two frequencies with considerable bandwidth difference.

Regarding the question on the effect of other frequencies, we had simulated different frequency excitations, but did not include in the paper. In fact, as the reviewer had queried, we too had the same question to see if the demodulation effect is visible with other AC frequency excitations. In the below figures, we show the effect of demodulation with different frequency combinations, thus proving the effect of demodulation with other excitation frequencies.

**Comment 3**- the demodulation effect should be better described.

**Reply:** Thank you for the comment. We have added more details to the demodulation effect, in Section 3.3 (in Page 14, from lines 278 -- 305).

**Comment 4-**Fig.9: the two scale are difficult to be distinguished (the one for electric and the one for magnetic field).

**Reply:** Thanks for the comment. We have added details of it in the figure 9 caption.

**Comment 5-** what is the application on electrical stimulation you are thinking of?

**Reply:** Electrical stimulation is the primary established method for modifying the operations of biological cells or organs as a treatment approach. Its applications are vast, encompassing the treatment of neural disorders such as Alzheimer's, Parkinson's, sleep apnea, as well as in brain-machine interfaces, spinal cord injuries, cochlear implants, and more. The current technology in use is a scaled version of stimulation derived from already established cardiac pacemakers. Research into new stimulation methodologies is ongoing, exploring the use of micro-coils, nanoparticles, optogenetics, and more. In this context, we introduce a novel, controllable stimulation platform designed to precisely target cells (specifically neurons) for creating sensations and paving the way for other future applications.

**Comment 6-** the structure of the paper is somewhat difficult to be read, why many of the results are in the discussion and analysis section and not in the results? it is not clear what is considered a true result and what an analysis. probably these two sections should be rewritten to clearly distinguish what is the new contribution of the paper.

**Reply:** Thank you for your feedback. We have revisited and updated the paper to enhance its clarity and structure.

**Reviewer #2:**

*We would like to express our sincere gratitude for the time and effort you dedicated to reviewing our paper. Your insightful comments have been invaluable. We have made the necessary modifications to the paper and addressed your feedback. Below, you'll find a detailed summary of the review comments along with our responses. We hope this revision meets your expectations and strengthens the quality of our work. Once again, thank you for your constructive feedback, and we look forward to any further suggestions or recommendations.*

**Comment 1:** The manuscript describes a novel magnetoelectric (ME) microdevice design (through in silico modelling) for wireless cellular stimulation therapy. Although this paper addresses an interesting topic, it is not publishable in its current form. In particular, it is very hard for the reader to follow the argument. The language and the structure of the manuscript are sometimes incomprehensible. I suggest a complete rewriting and language editing to enable a proper comprehension and revision.

**Reply:** Thanks for the comment. We have rewritten the entire paper, with better division of sections and subsections. Some of the sections required changes to the model after a few simulations. For example, we set a pre-biased condition at lower DC bias field, when simulating the magnetostrictive core. After we added the Piezo shell and simulated, we found that an optimum pre-bias condition to be 293mT. We then performed the previous simulation with the optimum bias value and analyzed the results.

**Comment 2:** Here some of the main criticisms: The state of the art related to the magnetoelectric materials and their use in biomedicine is lacking. Materials and Methods section are superficial: many details about the Comsol Model are missing. "Eigen mode" analysis, which seems to be the core of the manuscript, is described only in "Discussion and Analysis" section (not mentioned before). Some results, and the related figures, are presented for the first time in the "Discussion and Analysis" section. In any case, too many figures are presented (15), while most of them should be moved in the Supplementary Material. Most importantly, the novelty and advantages of the presented approach respect the state of the art are not clear.

**Reply:** Thanks for the comments. We have now added more references for ME. Moreover, ME and medical use is a growing research field with more articles and publications. We have now rewritten all the sections, including the Materials and Methods (Section 2). We have added Tables (1, 2 and 3), Figure 3, showing the modelling method we followed. As we explained before, the sections are now clear with the details and the results (Section 3), followed by the discussion section (Section 4). We were not able to move many figures to the supplementary file, because, we had to refer to certain figures and results and we felt it would be confusing for the reader to refer it every time in the supplementary file.

Regarding the novelty of the work, we have explained the need for two sources and to show the proof-of-concept of frequency demodulation. The novelty is a battery free ME device that could act as a frequency demodulator under physiological conditions. The Eigen mode operation amplifies the demodulated signal amplitude. We wanted to show both these hypotheses. Thus, we followed the steps mentioned in the Methods (Section 2).
