# Supplementary material for: Multiphysics simulation of magnetoelectric micro core-shells for wireless cellular stimulation therapy via magnetic temporal interference
Source: PLoS One. 2024 Jan 25;19(1):e0297114. doi: 10.1371/journal.pone.0297114 (PMC10834063; doi:10.1371/journal.pone.0297114)
Supplement: Attachment — Submitted filename: Support Letter--PONE-D-23-33833.docx [file pone.0297114.s002.docx]

Support Letter for Submission titled:

**Multiphysics simulation of magnetoelectric micro core-shells for wireless cellular stimulation therapy via magnetic temporal interference**

Dear editors,

We recently submitted our work to PLOS-ONE (PONE-D-23-33833) and have since received the review letter. We sincerely appreciate the valuable feedback provided and the time reviewers had spent. The comments were instrumental in helping us identify and address issues within our paper. We have thoroughly revised the manuscript and are now resubmitting it. Below, please find a detailed response to each review comment and the corresponding changes made in the current version.

**Reviewer #1:**

*We would like to express our sincere gratitude for the time and effort you dedicated to reviewing our paper. Your insightful comments have been invaluable. We have made the necessary modifications to the paper and thoroughly addressed your comments. Below is a detailed breakdown of the review comments alongside our responses. We hope that this revised version meets your expectations and further elevates the quality of the research presented.*

**Comment 1**: - The rationale for employing MTI signals for neural stimulation should be clarified early in the manuscript. Comparative simulations with both MTI and single-frequency magnetic fields could illustrate the advantage of MTI in generating larger electric fields around the ME particles.

**Reply:** Thanks for the comment. The rationale for using MTI is introduced in the introduction, which we have rewritten completely. Lines 98 – 100, and lines 108 – 117 explain the idea behind using MTI with ME particle mediation. The subsequent paragraphs (lines 119 – 127) also detail the MTI and the use of low frequency interference signal for direct electrical stimulation.

The main goal of using MTI is not to create a larger magnetic field but to create signal interference at the ME position that appears as a modulated wave or beating wave. We have provided comparative simulation to show the absence of frequency demodulation in an air medium. Moreover, we have also showed that the combined nonlinearity of ME material (specifically magnetostrictive core) and the beating wave creates low frequency counterpart on the piezo shell for direct electrical stimulation of cells (shown in Section 4.3, through the Hodgkin-Huxley model for generating neural spikes). We note that, using a single frequency would create high frequency deformation and thermal effects, while dual frequency or multiple frequencies would stimulate difference frequencies or other harmonics that can interact with the cells.

In subsequent papers, experiments to quantify the electrical field, through MTI and single frequency magnetic fields, and to validate the current simulation results, will be explored.

**Comment 2**: - The literature review in the introduction does not seem exhaustive.?

**Reply:** Thanks for the comment. We have rewritten the literature review with newer references and additions to align with the concept.

**Comment 3**- Specific comments on the manuscript:

• Line 64: Reference 16 appears misplaced as it does not pertain to implantable micro-coils as suggested.

The comment is rectified, as the whole literature survey is rewritten.

• Line 72: The claim of novelty in the magnetoelectric Multiphysics behavior should be substantiated. While similar works are cited later (e.g., Ref. 210), these should be integrated into the discussion at this point to establish the context of novelty effectively.

Comment is rectified, as the whole literature survey is rewritten. Magnetoelectric Multiphysics behaviour is not a novelty. The novelty lies on particle nonlinearity and application with MTI signaling, particle dimensions optimization and, additionally, the direct biological stimulation. This is clarified in the revised version.

• The practicability of the proposed ME particles is questionable given the requirement for two large coils on either side. A discussion on how this setup could be realistically implemented in a clinical setting is necessary.

In our paper, we show the concept design and not the practical development of the coils. We know that this can be two coils as in the model for in-vitro petri dish tests or two coils on the same plane to penetrate the magnetic field into the biological medium. The size of the coils is also relative to the penetration depth and the applied frequency. Therefore, the main aim is to show that the frequency demodulation serves as the system's output for cell stimulation. To ensure that the demodulation source is the magnetostrictive material (shown in Fig 7 and 8) and not the electromagnetic interference, we made the computations to support the idea. Depending on the distance, the intensity of the signals could also be controlled to match the desired magnetic field density.

• Incorporating an analysis of thermal effects within the current study would greatly enhance the research's value, especially given the significant concern of tissue temperature increases due to a large (100µm) ME particle.

Thank you for your insightful comment. We acknowledge that we have not extensively explored this broad area. To model the thermal effects accurately, it is essential to have access to data on the high-frequency performance of magnetostrictive components, including the ME coupling factor and the hysteresis loop of the material. Unfortunately, this information is not available at this stage. However, we wish to clarify that at high frequencies, such as in the 100 MHz range, the hysteresis loop, which is a primary source of thermal effects, will likely appear as a closed area loop, implying reduced thermal effects at higher frequencies. This aspect still requires further validation at the specific frequency.

At high frequencies, the primary heating source is eddy current, with the conductivity loss in the magnetic core being a dominant factor. The small size of the particles limits the impact of eddy currents on the surface, while the large surface-to-volume ratio facilitates heat transfer. Additionally, the skin depth, indicating the penetration of the magnetic field into the particle, is on the order of 20-30 microns, that is, in the order of particle size, showing the magnetic field penetration to the core of the particle for maximum deformation. These factors collectively aid in controlling thermal effects. However, due to our model's lack of fundamental data, we plan to conduct practical experiments to ensure a comprehensive and consistent study.

**Comment 3**- Minor Points for Revision:

1. The term 'microdevices' used to describe the ME particles could be misleading since the technology lacks electronic components and the broader scientific community typically refers to such entities as micro/nanoparticles.

Thank you. The definition of ‘devices’ might be misleading. We have changed this nomenclature to either particles, or composites, or core-shells.

2. **Specific comments on the manuscript:**

Line 60: The manuscript could acknowledge the existence of novel TMS systems capable of deep tissue layer stimulation.

Thank you. We have acknowledged it. Line 44, reference 16 is added for the same.

Line 221: The assertion made here requires substantiation with a relevant reference.

Changed. Reference and the specific table in the reference supporting the statement, are cited.

Line 504: The term "conventional magnetic temporal interface" seems to confuse "interface" with "interference." Moreover, a definition or citation for what constitutes conventional MTI is necessary for clarity.

Spell mistake corrected.

3. In Figure 3, it would be beneficial to specify the distance between the coil and the ME particle.

We have changed the figure (Fig. 2) to show the distance between the coils. In the explanation for the figure, we have added the distance from each of the coils to the ME particle (both the coils are at an equal distance from each other and the ME particle).

**Reviewer #2:**

*We would like to express our sincere gratitude for the time and effort you dedicated to reviewing our paper. Your insightful comments have been invaluable. We have made the necessary modifications to the paper and addressed your feedback. Below is a detailed summary of the review comments along with our responses. We hope this revision meets your expectations and strengthens the quality of our work. Once again, thank you for your constructive feedback, and we look forward to any further suggestions or recommendations.*

**Comment 1**: - We need ordered electric signals of ME nanoparticles to stimulate certain neurons. However, the polarization of the piezoelectric response depends on the poling directions (self-poled or externally poled). It means that the particle's electric response could cancel each other if they are in opposite poling conditions. So the key problem is how many particles is needed for stimulation of one neuron. If only one ME particle is needed, then it maybe useful. If more than one particles is needed, the accumulation effect of all particles should be carefully calculated and simulated.

**Reply:** Thank you for your insightful comment. When considering the interaction of a single particle with biological cells, it is important to note that the volume of the magnetostrictive material remains constant during the deformation process. This means the particle expands in one direction while simultaneously compressing in another. This assumption is particularly valid for the fundamental resonance mode of the particle, where the deformation results in charge distribution over the piezoelectric material. This charge distribution is then introduced into the biological medium, which possesses a certain low-frequency conductivity capable of creating current loops around the particle's surface. The resulting current extends beyond the particle into the medium, potentially interacting with neurons.

Due to the large size of the particle, submicron deformations are possible, allowing a single particle to act as multiple local electrodes. This leads to a situation where some of these 'electrodes' may cancel each other out, while others remain active and capable of stimulating cells. Additionally, the distribution of these 'hotspots' on the particle surface is not uniform, suggesting that a stochastic model might be able to predict the cells' reactions to the field more accurately.

When multiple such particles are present in a tissue, we anticipate individual particle interactions with neurons. However, this is a complex process, so the ultimate development of this technology will likely require a learning algorithm with a feedback loop from the cells. This algorithm would rectify and adjust the system to evoke a programmed action potential effectively.

**Comment 2**: - I also worry about the safety of this therapy. Particles, although small, usually cause unpredictable and undesirable problems for biological tissues. Many of them could result in cancer. How to solve this problem in this therapy.

**Reply:** A prevalent concern when utilizing such particles, particularly given their micro-size, is their potential to become dislodged and enter the bloodstream. To mitigate this risk, anchoring these particles within a structure is crucial to ensure stable interaction with cells. Implementing measures to secure these micro-particles is essential. Extensive research, including toxicology studies and post-medication analyses, has been conducted that should be referenced and adapted for the practical application of these particles in humans. One promising strategy is incorporating the particle into a hydrogel, using it as an intermediary agent for cell interaction.

**Reviewer #3:**

*We would like to express our sincere gratitude for the time and effort you dedicated to reviewing our paper. Your insightful comments have been invaluable. We have made the necessary modifications to the paper and addressed your feedback. Below is a detailed summary of the review comments along with our responses. We hope this revision meets your expectations and strengthens the quality of our work. Once again, thank you for your constructive feedback, and we look forward to any further suggestions or recommendations.*

**Comment 1**: -When applying AC magnetic field stimulation, it is recommended to address electromagnetic radiation safety thresholds. Authors also need to consider the attenuation of high-frequency AC magnetic fields within human tissue.

**Reply:** Thank you for your comment. The primary objective of our paper is to elucidate the interaction between the particle and magnetic fields. The frequencies applied in our study are exemplary selections, intended to demonstrate this interaction. For the final design, the choice of frequencies will be meticulously tailored based on the size of the fabricated particle and the specific magnetic field intensity required for stimulation therapy (preferably will be shifted to the range 6-20 MHz). As noted in the paper, the magnetic field in our experiments is generated by a closed-loop current path (coil). In this setup, the intensity of the magnetic field, represented by the Poynting vector components, plays a crucial role in power transfer. While biological tissues are inherently non-magnetic, the alternating magnetic field used in our experiments induces lower electric field. The main thermal effect will remain with the coil structure, which is external to the biological tissues. Notably, the magnetic field has a low loss effect on the tissues, making it a more dominant factor in our study. The use of the coil and magnetic field are key to creating low thermal loss in the tissues.

**Comment 2**: -The polarization direction of the piezoelectric crystal should be clearly indicated. It would be beneficial to discuss the impact of magnetic field stimulation from different directions on the output electric field.

**Reply:** Thanks for the comment. We have mentioned the polarization direction of the piezo shell (Line 205). Regarding the magnetic field, the material property of the magnetostrictive layer is non-linear isotropic, meaning that the magnetic field intensity depends on the operation of the core in its Eigenmodes, rather than the direction of the applied external field. For planar structures, the direction of magnetic field application will decide the definition of resonant modes and the operation of the magnetostrictive layer in length, width, or thickness modes (for more details on this, the reviewer could refer to Ref. 51, in the main document). We note that the carrier frequency, field direction, and intensity offer flexibility in the programmable targeting of different neurons in the vicinity of the particle.

**Comment 2**: -The material properties of MetGlas seem to be incomplete, particularly regarding the initial magnetic permeability.

**Reply:** I am assuming the reviewer is asking about the Initial Magnetic Susceptibility. We have included the material property in Table 1.
